# Clinical factors that influence the outcome of selective devascularization in the treatment of portal hypertension

**DOI:** 10.18632/oncotarget.9641

**Published:** 2016-05-26

**Authors:** Cheng-Lin Lu, Ya-Juan Cao, Hao Cheng, Yi-Ming Pan, Shan-Hua Bao, Min Xie

**Affiliations:** ^1^ Department of General Surgery, Nanjing Drum Tower Hospital, The Affiliated Hospital of Nanjing University Medical School, Nanjing 210008, Jiangsu Province, China

**Keywords:** selective devascularization, variceal hemorrhage, portal hypertension, clinical factors

## Abstract

There is a high incidence of death due to variceal hemorrhage in patients with portal hypertension. Factors to consider when choosing selective devascularization in the treatment of variceal hemorrhage remain a controversy. This study aims to generate the prevalent clinical risk factors that affect the outcomes of selective devascularization procedures. Elucidating these features may guide future treatment of esophageal varices in patients with portal hypertension. We retrospectively analyzed medical records of 455 patients who underwent selective devascularization procedures in our center. Patients were subject to splenectomy, selective devascularization with or without esophageal transection. The mode of surgery recurred in comparable rates in both the group with major complications postoperatively (high-risk group which consisted of 63 patients) or the group without major postoperative complications (low-risk group, 392). Risk factors that negatively influenced outcomes of surgery include severe symptoms (89% in high risk group and 71% in low risk group), large volume of blood loss in the hemorrhage before surgery (81% in high risk group and 16% in low risk group), sever liver cirrhosis (83% in high risk group and 67% in low risk group), previous endotherapy, prolonged prothrombin time, and poor liver function. Selective devascularization is a feasible option to treat variceal hemorrhage in patients with portal hypertension.

## INTRODUCTION

Portal hypertension is a significant complication of liver disease that presents with many challenging medical consequences, such as variceal hemorrhage, which is the leading cause of death in patients with portal hypertension [1]. Common sources of portal hypertension worldwide are hepatic cirrhosis and hepatic schistosomiasis [2]. Alcoholic and viral cirrhosis constitute most cases of patients with liver disease in Western countries [3]. Hepatitis B and schisotomiasis are the other, more endemic sources of portal hypertension in the Eastern regions including Southeast Asia and the Middle East [4]. An increasing incidence of liver cirrhosis worldwide is being caused by hepatitis C [5]. The morbidity and mortality of life-threatening emergency that could result from portal hypertension, particularly acute bleeding from esophageal varices, reduce the quality of lives of affected patients globally.

A variety of procedures have been developed to manage variceal bleeding; however none are proven to be as efficacious as desired. The risk of hemorrhage increases as the measure of portal hypertension, the hepatic venous pressure gradient (HVPG), exceeds 10 mmHg [2]. The annual incidence of first variceal hemorrhage can be up to 15% and has a significant mortality of 7%–15% [6–9]. Approximately 60–70% of patients will have recurrent variceal bleeding within a year of their first episode, and majority of these patients will die if left untreated [9–11]. Therefore effective secondary prophylaxis is essential in reducing mortality from repeat episodes. The most ideal treatment is liver transplantation, which reduces recurrent hemorrhage while maintaining liver function. However, not all patients are candidates for transplant and there are significant wait times and financial impacts associated with the operation [12]. Currently accepted standard treatments include pharmacotherapy and endotherapy. Non-selective beta blockers and endoscopic variceal ligation, which is presently selected over sclerotherapy, remain the preferred regimen to prevent rebleeding [3]. Transjugular intrahepatic portosystemic shunts (TIPS) are implemented in patients who have Child-Turcotte-Pugh (CTP) A or B cirrhosis with repeat hemorrhages despite pharmacologic and endoscopic therapy, but its effectiveness is variable [4, 13–21]. A substantial rate (40–50%) of stenosis and thrombosis limits the use of TIPS [22].

It has been reported that techniques such as the modified Sugiura procedure are effective in treating variceal hemorrhage [23–26]. The original Sugiura procedure consisted of paraesophagogastric devascularization and esophageal transection through the chest and abdomen [27]. Though applicable, the technique was extremely complex, complications were dangerous, and results were inconsistent. It was simplified into the modified Sugiura procedure, which is comprised of paraesophagogastric devascularization and esophageal transection in the abdomen [22, 28]. These surgical techniques may become increasingly indicated in CTP A or B patients as an alternative to sclerotherapy or endoscopic ligation [29]. Selective devascularization 1) does not affect liver function, 2) reliably reduces the risk of rebleeding, and 3) decreases Free Portal Pressure (FPP) to prevent genesis of new capillaries [28]. Further optimization is needed to explore these options. This study aims to explore the various therapies and their outcomes at our treatment facility in order to guide future treatment scenarios.

## RESULTS

### Patient characteristics

A total of 500 patients were enrolled in this study. Due to some incomplete medical records, the final analysis consisted of 455 individuals. In these patients, 63 cases had complications and were stratified into the high-risk group. 392 cases had no complication and were classified as low-risk. One patient died of Acute Respiratory Distress Syndrome (ARDS) immediately after surgery in the intensive care unit (ICU).

Male gender was the predominant sex in the incidence of portal hypertension, and represented 41 cases (65%) of patients in the high-risk group and 237 (60%) cases in the low-risk group. In the high-risk group, 59 (89%) cases had severe complaints including hematemesis and black stool (melena); 7 (11%) cases had mild complaints such as fatigue and others. The high- and low- risk groups demonstrated no difference in age, gender, and type of previous liver disease. Among the high-risk group, 81% (51 cases) had total bleeding of more than 1000 ml, while only 16% (62 cases) had bleeding of over 1000 ml in the low-risk group before surgery. There was statistical significance in the difference between the number of patients complaints and volume of bleeding between the high- and low-risk groups. However, there was no difference concerning the number of bleeding episodes in these two groups of patients. These results indicate that the severity of patients' complaints and the severity of bleeding before surgery might contribute to the outcomes of selective operation procedure.

It was not surprising to note that most of the patients in both high- and low-risk groups suffering from portal hypertension had a history of liver disease with hepatitis B, as that is a major cause of liver disease in China. Sources of liver disease were not directly related to the complications of surgery. However, 79% (50 cases) of patients in high-risk group, and 68% (274 cases) of patients in the low-risk group had liver cirrhosis, indicating that liver cirrhosis might contribute to the complication of surgery. The patients characteristics are summarized in Table 1.

**Table 1:** Patients characteristics

Patients (*n* = 455)	High-risk (*n* = 63)	Low-risk (*n* = 392)	*P* value
**Age**			
Range	19–74	5–79	
Average	50	47	0.7931
**Gender**			
Male	41 (65%)	237 (60%)	0.8332
Female	22 (35%)	155 (40%)	0.8376
**Complains**			
Severe	56 (89%)	278 (71%)	0.0331*
Mild	7 (11%)	109 (29%)	0.0272*
**Volume of bleeding**			
(before surgery)			
>1000 ml (case, %)	51 (81%)	62 (16%)	0.0038*
≤ 1000 ml (case, %)	12 (19%)	321 (84%)	0.0079*
**Episodes of bleeding**			
> 2 (case, %)	18 (29%)	107 (27%)	0.0632
≤ 2 (case, %)	45 (71%)	285 (73%)	0.0785
**Liver disease**			
Alcoholic	2 (3%)	5 (1%)	0.0874
Hepatitis B	43 (68%)	234 (60%)	0.0675
Hepatitis C	1 (1%)	43 (11%)	0.0527
Others	18 (28%)	110 (28%)	0.0946
**Liver cirrhosis**			
Yes	52 (83%)	264 (67%)	0.0436*
No	11 (17%)	128 (33%)	0.0365*

* indicates statistical significance.

### Factors potentially affect selective devascularization

Endotherapy and pharmacotherapy are the current accepted first-line treatment for variceal bleeding in patients with portal hypertension [3]. We investigated whether previous treatment with endotherapy correlates with the incidence of complication. We found that patients who did not receive endotherapy before selective devasculariation had a lower chance of producing a complication, although this might implicate that variceal bleeding is less severe in this group of patients.

Three types of selective devascularization were performed: A. Selective devascularization with esophageal transection; B. Paraesophagogastric devascularization without esophageal transection; and C. Splenectomy. About 70% of patients in both group received selective devascularization with esophageal transection, 20% of patients received paraesophagogastric devascularization without esophageal transection and less than 10% of patients received splenectomy. The mode of surgery did not affect the treatment outcomes of patients.

The portal branches were retained during surgery whenever possible. This effort resulted in a lower incidence of complications. The volume of bleeding and the volume of blood transfusion during surgery did not affect the outcome of selective devascularization.

The free portal pressure (FPP) was measured before and after operation. It did not differ a lot before the surgery in the high- and low-risk groups. However, there was a lower FPP after surgery in the low-risk group. Interestingly, we noted that less complication occurred when patients had a greater FPP.

Factors that potentially affected selective devascularization are summarized in Table 2.

**Table 2:** Factors affect selective devascularization

Patients (*n* = 455)	High-risk (*n* = 63)	Low-risk (*n* = 392)	*P* value
**Endotherapy**			
Yes	15 (24%)	40 (10%)	0.0410*
No	48 (76%)	352 (90%)	0.0375*
**Mode of surgery**			
A	43 (68%)	292 (74%)	0.0648
B	15 (24%)	66 (17%)	0.0752
C	5 (8%)	34 (9%)	0.0896
**Retain portal branches**			
Yes	47 (74%)	367 (93%)	0.0231*
No	16 (26%)	21 (7%)	0.0129*
**Volume of bleeding**			
(during operation)			
> 1000 ml	19 (30%)	94 (24%)	0.0568
≤ 1000 ml	44(70%)	298 (76%)	0.0632
**Blood transfusion**			
> 1000 ml	22 (35%)	119 (30%)	0.0679
≤ 1000 ml	41 (75%)	273 (70%)	0.0731
**FPP (cm H_2_O)**	44.3	44.6	0.0872
Pre-operation	38.1	35.9	0.0389*
Post-operation	7	8.7	0.0271*
Difference of FPP	1.45	1.46	0.0982
**Diameter of portal vein (cm)**			

Type of surgery:

A. Selective devascularization with esophageal transection

B. Paraesophagogastric devascularization without esophageal transection

C. Splenectomy

* indicates statistical significance.

### Pre- and Post-operative parameters

Since the patients' general condition and liver function can affect the outcome of selective devascularization, we performed routine blood examination and liver function tests. The average values of the tests are listed in Table 3. We found that blood AST (Aspartate transaminase) was significantly higher in the high-risk group than that in the low-risk group. There was longer prolonged prothrombin time in the high-risk group.

**Table 3:** Pre- and Post-operative parameters

Patients (*n* = 455)	High-risk (*n* = 63)	Low-risk (*n* = 392)	*P* value
**Before surgery**			
WBC (×10^9^/L)	2.7	2.2	0.5327
Hb (g/L)	9.0	9.3	0.8764
Platelet (×10^9^/L)	50	57	0.7632
ALT (U/L)	46.7	33.7	0.6829
AST (U/L)	51.4	40.9	0.0412*
ALB	34.8	36.2	0.0738
GLB	28.9	27.9	0.0824
TB	29.0	24.8	0.0689
DB	10.9	9.5	0.0721
**One week after**			
WBC (×10^9^/L)	12.5	11.6	0.0652
Hb (g/L)	10.5	11.3	0.0872
Platelet (×10^9^/L)	166.5	221.2	0.0571
ALT (U/L)	28.2	30.4	0.0976
AST (U/L)	23.1	35.9	0.0568
ALB	38.3	34.7	0.0875
GLB	26.0	26.6	0.1899
TB	24.3	24.0	0.0978
DB	9.0	9.9	0.0897
**PT** (second)	16.0	15.7	0.0813
**Prolonged PT**	6.5	0.7	0.0042*
**Liver function**			
CTP-A	34 (54%)	277 (71%)	0.0298*
CTP-B	24 (38%)	103 (28%)	0.0357*
CTP-C	5 (8%)	5 (1%)	0.0154*

* indicates statistical significance.

The Child-Turcotte-Pugh (CTP) score, which assesses the prognosis of chronic liver disease, was used to evaluate the patients liver function. We found that there were significantly fewer patients in the high-risk group whose liver function was CTP-A, indicating that liver function was a contributing factor for complication.

## DISCUSSION

Therapy for variceal hemorrhage due to portal hypertension has changed immensely over the years, yet the mortality remains high and urgency to optimize the treatment stays strong. Our treatment facility is nationally renowned for expert management of portal hypertension. There are a large number of patients seeking treatment of variceal hemorrhage in our center. Goal of therapy is to prevent rebleeding while maintaining liver function. In this case, liver transplantation meets all the objectives, but it is not a viable option for most patients. The current standard treatment of variceal hemorrhage is pharmacotherapy and endotherapy. TIPS is a more advanced, non-operative therapy to decrease variceal pressure and is increasingly popular but there are many contraindications (e.g. venous thrombosis) and there is a high rate of limiting complications [3, 30]. Therefore, alternative methods are required to treat variceal hemorrhage. To visualize the pathophysiology, varices form when the portal blood flow is met with resistance and the blood regurgitate in the opposite direction [31]. Therefore, terminating the blood supply to form these varices may prevent their increased blood flow and consequently, bleeding; this was the basis of the original Sugiura procedure [27]. Since then, it has been modified to accommodate diverse patient factors and nuances in their conditions. The study elucidates some of those factors that can influence clinical decision-making.

In this patient cohort, the major complaints were hematemesis and melena, while minor ones included those such as fatigue. As expected, the number of patient who have major complaints was higher in the high-risk group than that of the low-risk group, most likely due to the more sever varices they have experienced. The volume of blood resulting from the hemorrhage differed in both groups as well. 89% of the patients in the high-risk group had bleeding of more than 1000 ml compared with only 16% of patients in the low-risk group. This excess amount of bleeding could have additionally contributed to the severity of the patients' postoperative complications.

Source of liver disease (e.g. hepatitis) did not affect the outcome of the surgeries. Only higher percentage of patients presented with cirrhosis, signifying that cirrhosis may be a relevant factor to the complication of surgery. The study also examined the rate of previous endotherapy in both groups. 24% of patients in the high-risk group had received endotherapy in the past while only 10% had in the low-risk group. This difference was statistically significant, suggesting that previous endotherapy may have contributed to the outcome of surgery. Conversely, retaining portal branches resulted in a higher percentage of low-risk patients (93% vs. 74% in high-risk group).

Patients' laboratory tests were analyzed to further show objective results. Firstly, liver function is an understandable factor that would affect surgery outcome. 71% of patients in low-risk group and only 54% in high-risk group were CPT A patients, and the trend reversed as CTP grade worsened (CTP B: 28% in low-risk and 38% in high-risk; CTP C: 1% in low-risk and 8% in high-risk). The blood AST level was significantly increased in the high-risk group than in the low-risk group before the surgery, which is another indicator of liver function. Additional distinct observation in the high-risk group was the prolonged prothrombin time, another sign of liver failure as well as a measure of reduced coagulabilit [32]. which increases bleeding risk in patients.

Currently, surgical procedures may be indicated only in CTP A or B patients to resect veins and prevent variceal bleeding early while liver function is intact [33]. There are also some risk factors associated with rebleeding in selective devascularization. Incomplete devascularization, resulting portal hypertension gastropathy, and regeneration of the varices can contribute to increased rebleeding risk. Prospective, longer-term analysis is needed to better understand the impact of the procedure.

This retrospective analysis showed that the modes of surgery were operated at comparable rates in both groups, which shows that type of surgery did not influence the outcomes. Those who were not subject to esophageal transection may have had a procedure just as effective and safe with less morbidity associated with esophageal transection [22]. Furthermore, a previous meta-analysis showed that the incidence of encephalopathy is higher in patients who have previously had a shunt than in those who underwent devascularization [34]. Therefore, selective devascularization may be a viable alternative in patients with previously failed therapies.

The most notable limitation of this study is that this was a single center retrospective analysis and thus several confounding variables are likely to have affected the results. A future study with a larger patient cohort from multiple centers could provide some standards for the decision-making in treating patients with portal hypertension.

## CONCLUSIONS

Various patient characteristics influence the result of procedures. Risk factors that may be negative predictors of selective devascularization outcomes are severe symptoms such as hematemesis, large volume of bleeding during the episode, liver cirrhosis, previous endotherapy, prolonged prothrombin time, and poor liver function. It was concluded that selective devascularization is an effective alternative therapy to treat variceal hemorrhage from portal hypertension in those who are not candidates for shunts or transplant.

## MATERIALS AND METHODS

### Patients

This study was approved by the Internal Review Board of Nanjing Drum Tower Hospital, the affiliated hospital of Nanjing University Medical School. 500 patients with portal hypertension who underwent selective devascularization in the Department of General surgery, Nanjing Drum Tower Hospital, from January 1995 to December 2014 were included in the present study. The clinical data from the medical records of these patients were retrospectively analyzed. Among these patients, there were 182 females and 318 males. The average age of patients was 48 years old (range 5–79 years old).

The patients were divided into high-risk group and low-risk group according to their postoperative complications. The patients were considered high-risk if they had one of the following severe complications within 6 months after surgery: liver failure, kidney failure, multi-organ function failure, severe gastrointestinal bleeding, bleeding into abdominal cavity, portal thrombosis, pleural effusion, abdominal ascites, liver abscess, encephalopathy, pulmonary infection, other liver dysfunction-related diseases, or death. Patients with no postoperative complication were considered as low-risk. For chief complaints, we defined severe complaints as hematemesis and black stool (melena); mild complaints as fatigue and comparative others. Definition of liver disease included all sources of liver disease, such as alcoholic cirrhosis, hepatitis B, hepatitis C, and others. The grade of liver cirrhosis was evaluated using Child-Turcotte-Pugh (CTP) classification.

### Operation indications

The operation indications include: failure of medical treatment, endoscopic therapy is invalid, repeated hemorrhage, hypersplenism, and the patient's liver function should be Child-Turcotte-Pugh A or B.

### Operation procedures

**Figure 1:**
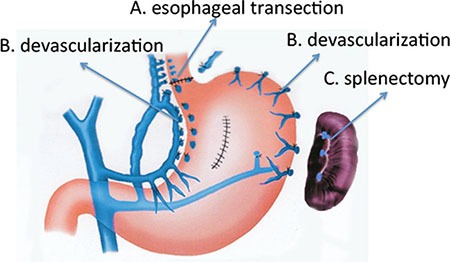
The schema showed the details of surgical procedures

Surgical procedure was selected based on patients' general condition. 335 patients received selective devascularization with esophageal transection. 81 patients received paraesophagogastric devascularization without esophageal transection. 39 patients received splenectomy. The details of the procedures are shown in Figure 1.

### Patient follow-up

The patients were evaluated at 6 months, 1year, 2 years and 5 years. The follow-up included assessment of patients' general condition, the quality of life, and re-bleeding rates.

### Statistical analysis

Statistical analysis was performed using the software SPSS15.0. Qualitative data were analyzed by the *X*^2^ test and Fisher's exact test. Quantitative data were analyzed by the *t*-test. Statistical significance was considered if *P* < 0.05.

## References

[1] Bosch J, Abraldes JG, Berzigotti A, Garcia-Pagan JC (2008). Portal hypertension and gastrointestinal bleeding. Semin Liver Dis.

[2] Berzigotti A, Seijo S, Reverter E, Bosch J (2013). Assessing portal hypertension in liver diseases. Expert Rev Gastroenterol Hepatol.

[3] Garcia-Tsao G, Sanyal AJ, Grace ND, Carey W (2007). Practice Guidelines Committee of the American Association for the Study of Liver Diseases; Practice Parameters Committee of the American College of Gastroenterology. Prevention and management of gastroesophageal varices and variceal hemorrhage in cirrhosis. Hepatology.

[4] El-Tawil AM (2012). Trends on gastrointestinal bleeding and mortality: where are we standing?. World J Gastroenterol.

[5] Lozano R, Naghavi M, Foreman K, Lim S, Shibuya K, Aboyans V, Abraham J, Adair T, Aggarwal R, Ahn SY, Alvarado M, Anderson HR, Anderson LM (2012). Global and regional mortality from 235 causes of death for 20 age groups in 1990 and 2010: a systematic analysis for the Global Burden of Disease Study 2010. Lancet.

[6] Villanueva C, Piqueras M, Aracil C, Gómez C, López-Balaguer JM, Gonzalez B, Gallego A, Torras X, Soriano G, Sáinz S, Benito S, Balanzó J (2006). A randomized controlled trial comparing ligation and sclerotherapy as emergency endoscopic treatment added to somatostatin in acute variceal bleeding. J Hepatol.

[7] Augustin S, Altamirano J, González A, Dot J, Abu-Suboh M, Armengol JR, Azpiroz F, Esteban R, Guardia J, Genescà J (2011). Effectiveness of combined pharmacologic and ligation therapy in high-risk patients with acute esophageal variceal bleeding. Am J Gastroenterol.

[8] Abraldes JG, Villanueva C, Bañares R, Aracil C, Catalina MV, Garci A-Pagán JC, Bosch J (2008). Spanish Cooperative Group for Portal Hypertension and Variceal Bleeding. Hepatic venous pressure gradient and prognosis in patients with acute variceal bleeding treated with pharmacologic and endoscopic therapy. J Hepatol.

[9] Garcia-Tsao G, Bosch J (2010). Management of varices and variceal hemorrhage in cirrhosis. N Engl J Med.

[10] Graham DY, Smith JL (1981). The course of patients after variceal hemorrhage. Gastroenterology.

[11] Burroughs AK (1993). The natural history of varices. J Hepatol.

[12] Garrett KO, Reilly JJ, Schade RR, van Thiel DH (1988). Bleeding esophageal varices: treatment by sclerotherapy and liver transplantation. Surgery.

[13] Cabrera J, Maynar M, Granados R, Gorriz E, Reyes R, Pulido-Duque JM, Rodriguez SanRoman JL, Guerra C, Kravetz D (1996). Transjugular intrahepatic portosystemic shunt versus sclerotherapy in the elective treatment of variceal hemorrhage. Gastroenterology.

[14] Cello JP, Ring EJ, Olcott EW, Koch J, Gordon R, Sandhu J, Morgan DR, Ostroff JW, Rockey DC, Bacchetti P, LaBerge J, Lake JR (1997). Endoscopic sclerotherapy compared with percutaneous transjugular intrahepatic portosystemic shunt after initial sclerotherapy in patients with acute variceal hemorrhage. A randomized, controlled trial. Ann Intern Med.

[15] García-Villarreal L, Martínez-Lagares F, Sierra A, Guevara C, Marrero JM, Jiménez E, Monescillo A, Hernández-Cabrero T, Alonso JM, Fuentes R (1999). Transjugular intrahepatic portosystemic shunt versus endoscopic sclerotherapy for the prevention of variceal rebleeding after recent variceal hemorrhage. Hepatology.

[16] Jalan R, Forrest EH, Stanley AJ, Redhead DN, Forbes J, Dillon JF, MacGilchrist AJ, Finlayson ND, Hayes PC (1997). A randomized trial comparing transjugular intrahepatic portosystemic stent-shunt with variceal band ligation in the prevention of rebleeding from esophageal varices. Hepatology.

[17] Merli M, Salerno F, Riggio O, de Franchis R, Fiaccadori F, Meddi P, Primignani M, Pedretti G, Maggi A, Capocaccia L, Lovaria A, Ugolotti U, Salvatori F (1998). Transjugular intrahepatic portosystemic shunt versus endoscopic sclerotherapy for the prevention of variceal bleeding in cirrhosis: a randomized multicenter trial. Gruppo Italiano Studio TIPS (G.I.S.T.). Hepatology.

[18] Pomier-Layrargues G, Villeneuve JP, Deschênes M, Bui B, Perreault P, Fenyves D, Willems B, Marleau D, Bilodeau M, Lafortune M, Dufresne MP (2001). Transjugular intrahepatic portosystemic shunt (TIPS) versus endoscopic variceal ligation in the prevention of variceal rebleeding in patients with cirrhosis: a randomised trial. Gut.

[19] Rössle M, Deibert P, Haag K, Ochs A, Olschewski M, Siegerstetter V, Hauenstein KH, Geiger R, Stiepak C, Keller W, Blum HE (1997). Randomised trial of transjugular-intrahepatic-portosystemic shunt versus endoscopy plus propranolol for prevention of variceal rebleeding. Lancet.

[20] Sanyal AJ, Freedman AM, Luketic VA, Purdum PP, Shiffman ML, Cole PE, Tisnado J, Simmons S (1997). Transjugular intrahepatic portosystemic shunts compared with endoscopic sclerotherapy for the prevention of recurrent variceal hemorrhage. A randomized, controlled trial. Ann Intern Med.

[21] Sauer P, Theilmann L, Stremmel W, Benz C, Richter GM, Stiehl A (1997). Transjugular intrahepatic portosystemic stent shunt versus sclerotherapy plus propranolol for variceal rebleeding. Gastroenterology.

[22] Zhang HY, Li WB, Ye H, Xiao ZY, Peng YR, Wang J (2014). Long-term results of the paraesophagogastric devascularization with or without esophageal transection: which is more suitable for variceal bleeding?. World J Surg.

[23] Jenkins SA, Shields R (1989). Variceal haemorrhage after failed injection sclerotherapy: the role of emergency oesophageal transection. Br J Surg.

[24] Mathur SK, Shah SR, Soonawala ZF, Karandikar SS, Nagral SS, Dalvi AN, Mirza DF (1997). Transabdominal extensive oesophagogastric devascularization with gastro-oesophageal stapling in the management of acute variceal bleeding. Br J Surg.

[25] Spence RA, Johnston GW (1985). Results in 100 consecutive patients with stapled esophageal transection for varices. Surg Gynecol Obstet.

[26] Terblanche >J (1989). The surgeon's role in the management of portal hypertension. Ann Surg.

[27] Sugiura M, Futagawa S (1984). Results of six hundred thirty-six esophageal transections with paraesophagogastric devascularization in the treatment of esophageal varices. J Vasc Surg.

[28] Gouge TH, Ranson JH (1986). Esophageal transection and paraesophagogastric devascularization for bleeding esophageal varices. Am J Surg.

[29] Tsukada K, Yoshida K, Hatakeyama K, Muto T (1997). Transthoraco-phrenic esophageal transection with paraesophago-gastric devascularization and splenectomy using a stapler. Hepatogastroenterology.

[30] Orozco H, Mercado MA, Martinez R, Tielve M, Chan C, Vasquez M, Zenteno-Guichard G, Pantoja JP (1998). Is splenectomy necessary in devascularization procedures for treatment of bleeding portal hypertension?. Archives of Surgery.

[31] Garcia-Pagan JC, Gracia-Sancho J, Bosch J (2012). Functional aspects on the pathophysiology of portal hypertension in cirrhosis. J Hepatol.

[32] Korte W, Clarke S, Lefkowitz JB (2000). Short activated partial thromboplastin times are related to increased thrombin generation and an increased risk for thromboembolism. Am J Clin Pathol.

[33] Selzner M, Tuttle-Newhall JE, Dahm F, Suhocki P, Clavien PA (2001). Current indication of a modified Sugiura procedure in the management of variceal bleeding. J Am Coll Surg.

[34] Yin L, Liu H, Zhang Y, Rong W (2013). The surgical treatment for portal hypertension: a systematic review and meta-analysis. ISRN Gastroenterol. 2013.

